# Current Status of In Vitro Models and Assays for Susceptibility Testing for Wound Biofilm Infections

**DOI:** 10.3390/biomedicines7020034

**Published:** 2019-04-30

**Authors:** Tania F. Bahamondez-Canas, Lara A. Heersema, Hugh D. C. Smyth

**Affiliations:** 1 Division of Molecular Pharmaceutics and Drug Delivery, College of Pharmacy, The University of Texas at Austin, Austin, TX 78712, USA; tania.bahamondez@utexas.edu; 2 Department of Biomedical Engineering, Cockrell Scholl of Engineering, The University of Texas at Austin, Austin, TX 78712, USA; Lheersema@utexas.edu; 3 The LaMontagne Center for Infectious Disease, The University of Texas at Austin, Austin, TX 78712, USA

**Keywords:** biofilms, chronic infections, in vitro models, viability assays

## Abstract

Biofilm infections have gained recognition as an important therapeutic challenge in the last several decades due to their relationship with the chronicity of infectious diseases. Studies of novel therapeutic treatments targeting infections require the development and use of models to mimic the formation and characteristics of biofilms within host tissues. Due to the diversity of reported in vitro models and lack of consensus, this review aims to provide a summary of in vitro models currently used in research. In particular, we review the various reported in vitro models of *Pseudomonas aeruginosa* biofilms due to its high clinical impact in chronic wounds and in other chronic infections. We assess advances in in vitro models that incorporate relevant multispecies biofilms found in infected wounds, such as *P. aeruginosa* with *Staphylococcus aureus*, and additional elements such as mammalian cells, simulating fluids, and tissue explants in an attempt to better represent the physiological conditions found at an infection site. It is hoped this review will aid researchers in the field to make appropriate choices in their proposed studies with regards to in vitro models and methods.

## 1. Introduction

**Figure 1:**
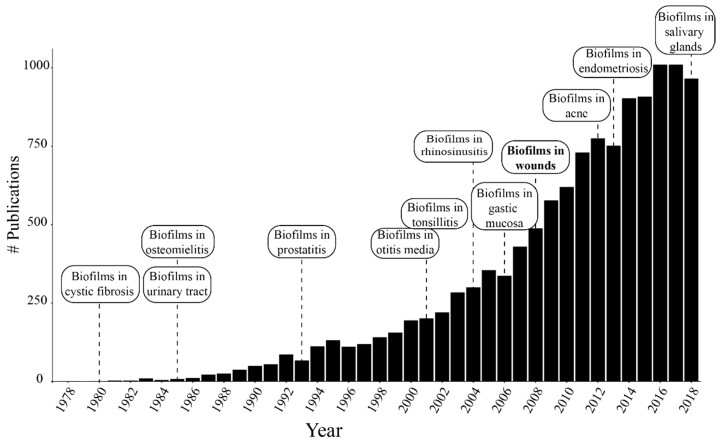
Number of publications per year with the word biofilms in the title of the article searched by Google Scholar. The labels indicate the dates where the evidence of biofilms in new tissues was published.

Biofilms are microbial communities that grow in aggregates and represent the predominant growth form for bacteria in nature, as compared with free-floating bacteria, known as the planktonic growth form [1]. However, most of our knowledge in clinical microbiology was built on studies of bacteria grown suspended in liquid media, which favors planktonic proliferation [2]. A new era of biofilm research began following the first observations of bacterial growth when attached to surfaces, the acknowledgement of biofilm infections in human tissues, and their relation to chronic infections [3]. The first reports described sessile bacteria growing attached to a surface and within a slimy substance [4]. The slimy substance, typically described as the biofilm extracellular matrix (ECM) or extracellular polymeric substances (EPS), is the hallmark of this life form [5]. Biofilms can adapt to different biotic and abiotic environments and resist changes in the environment due to the ECM. Oral bacterial plaque is probably the first recognized biofilm affecting human tissues [6,7]. Subsequent identification of biofilm-growing microorganisms on other mucosal surfaces has helped to elucidate the mechanisms of resistance and recurrence of several infections. Biofilms have been identified in a large variety of infections, including cystic fibrosis-associated respiratory infections [8], urinary tract infections [9], osteomyelitis [10], prostatitis [11], otitis media [12,13], periodontitis [14], tonsillitis [15], rhinosinusitis [16], gastric mucosa infections [17], chronic wounds [18], acne [19], endometriosis [20], and sialadenitis [21]. Figure 1 shows the development of biofilm research and evidence of the presence of biofilm infections in different tissues over the last four decades. Specifically, from 2008 to 2018 the number of publications in the area has doubled.

### 1.1. Biofilms in Chronic Wounds and Other Chronic Infections

Once bacteria successfully develop a biofilm, these infections usually become chronic and are extremely hard to eradicate. Biofilm matrix components, such as DNA and alginate, bacterial surface proteins, and quorum sensing (QS) molecules can induce an excessive inflammatory host response [22,23]. Subsequently, neutrophils accumulate around the biofilm and release oxygen species, proteinases, and other inflammatory factors while QS molecules induce upregulation of pro-inflammatory cytokines [24]. However, the ECM restricts the activity of these immune factors and, as a consequence, the continuous activation of the immune response damages the host tissue around the biofilm [25,26]. Also, biofilms act as a reservoir for planktonic bacteria that can spread and multiply, causing acute infections remotely. Therefore, biofilm infections are characterized by a chronic inflammatory response with recurrent acute episodes and resistance to antimicrobial therapy and host defenses. 

Chronic wounds, also described as *ulcers*, are defined as wounds that fail to heal within an expected order and time [27]. While acute wounds usually heal in about 4 weeks, chronic wounds can take more than 12 weeks, often re-occur, and commonly affect diabetic and elderly patients [28]. There are several factors that delay the normal wound healing process such as infections, and impaired vascularization and mobility [29]. The exudate released from the wound bed is a nutrient rich fluid that helps in the regeneration of wound tissue [30]. However, in chronic wounds the prolonged exposure of the host tissue and the exudate to the environment enhances the risk of acquiring infections. Infections of wounds can lead to delayed healing, amputation, sepsis, and death [31]. 

*Staphylococcus aureus, Enterococcus faecalis,* and *Pseudomonas aeruginosa* are among the most frequently found species in chronic wounds [18,32,33]. *P. aeruginosa* is an opportunistic gram-negative bacterium. It has recently been classified as a priority pathogen for the research and development of novel antimicrobial treatments due to its increasing antibiotic resistance and its relevance in health care-associated infections [34,35]. *P. aeruginosa* growing as biofilms have been found in chronic wounds but are also relevant in cystic fibrosis-associated lung infections. *S. aureus* is an opportunistic gram-positive bacterium that has gained recognition due to the increasing prevalence of the methicillin-resistant strain (MRSA) that can be acquired nosocomially [36].

The presence of biofilms in chronic wounds is now recognized as a factor to explain the impaired healing in these wounds [26,37,38]. The presence of biofilms was suspected due to the similarities between the sustained inflammatory response of chronic wounds with other biofilm infections over time [39,40]. James et al. provided the first evidence of the high prevalence of biofilms in chronic wounds. The study reported biofilms in more than 60% of the studied wounds, with a minor prevalence in acute wounds (only in 6%) [33]. These findings were confirmed by additional reports [18,41].

While *P. aeruginosa* and *S. aureus* are the most frequently isolated bacteria from chronic wounds, these organisms had to develop adaptations to co-exist. When co-cultured planktonically in vitro, *P. aeruginosa* inhibits *S. aureus* growth [42]. However, the formation of biofilms has shown not only the structure to allow their coexistence in the wound environment but also a benefit to both species [43,44]. These biofilms often appear nonrandomly distributed within wound locations [45]. In chronic leg ulcers, *P. aeruginosa* is localized in the deep regions of the wounds, while *S. aureus* is usually detected near the surface layer of the wound [46]. This differential distribution may be an adaptation for the coexistence of these biofilms within the same environment and also explains the underestimated prevalence of *P. aeruginosa* usually reported in chronic wounds. The study by Kirketerp-Moller highlighted these disparities when using different identification methods [18]. The study demonstrated the higher prevalence of *P. aeruginosa* biofilms in deeper regions of the wounds by advanced molecular techniques, suggesting the importance of the role of this microorganism in chronic wounds as compared to *S. aureus*. The identification of *P. aeruginosa* has been correlated with worse clinical outcomes in chronic wounds, such as an excessive inflammatory response, larger ulcer sizes, and a subsequently delayed healing [47]. As of 2012, chronic wounds accounted for over $1 billion in health care spending in the United States alone, and the estimated number of at-risk patients is only expected to increase [48]. Biofilm-focused treatments have shown promising results and improved wound healing [49], which confirms the impact of biofilm infections on the outcomes of chronic wounds.

Cystic fibrosis-related lung infections are another condition where *P. aeruginosa* biofilms are present. Respiratory disease is characteristic of cystic fibrosis and the main cause of morbidity and mortality [50]. In cystic fibrosis, the dysfunction of the transmembrane conductance regulator (CFTR) causes altered mucociliary clearance and a mucus layer forms that is more dehydrated, hyperosmotic, and viscous than in healthy patients [51]. This environment favors the accumulation and proliferation of bacteria. After the identification of *P. aeruginosa* growing as biofilms in the lungs of these patients, this chronic lung infection turned into the icon of biofilm-associated infections and *P. aeruginosa* became the model species for biofilm infection studies [52]. *P. aeruginosa* also causes persistent infections in non-cystic fibrosis respiratory diseases, such as bronchiectasis and chronic obstructive pulmonary disease [53,54], and in chronic rhinosinusitis [55]. The role of *P. aeruginosa* in urinary tract infections is minor compared to the diseases described above. However, it is one of the three most common pathogens isolated as biofilms from catheter-associated urinary tract infections [56].

### 1.2. Mechanism of Biofilm Resistance

Biofilms are known to be hundreds to a thousand times more resistant than planktonic bacteria to antimicrobials [57]. However, this resistance is not entirely explained by the rise of resistant strains—a current global concern recognized by the World Health Organization (WHO) [58]. In general, this high resistance has been explained by the protective role of the ECM (physical protection) and by the slow growth of microorganisms (phenotypic resistance) in addition to the mutant antimicrobial resistant strains (genotypic resistance). 

The role of bacterial communication during biofilm formation has been extensively discussed in an ongoing debate and remains unclear [59]. Bacterial signaling molecules (also known as quorum sensing (QS)) are known to control bacterial motility, biofilm formation and maturity, and expression of virulence factors by *P. aeruginosa* [60]. However, this signaling seems to be influenced by environmental conditions such as nutrient availability [61]. Still, investigational QS inhibitors have shown promising results preventing biofilm formation and reducing its virulence [62,63]. The role of QS signaling on the control of bacterial behavior has been recently revised [64].

#### 1.2.1. Extracellular Matrix (ECM)

**Figure 2:**
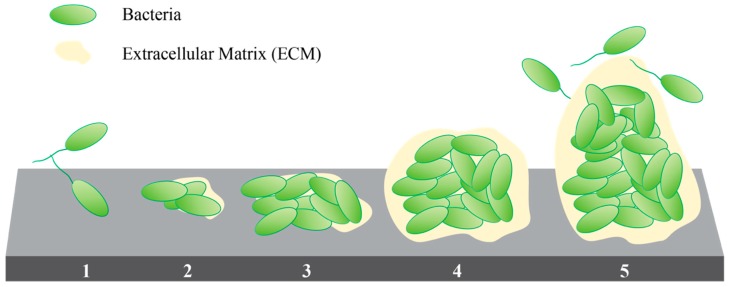
Stages of biofilm development: (1) Reversible attachment, (2) Irreversible attachment, (3) Maturation 1, (4) Maturation 2, and (5) Dispersion [76].

Biofilm development starts after the irreversible attachment of a planktonic cell that proliferates and initiates the secretion of ECM components (Figure 2) [65]. The ECM is a complex hydrated network of polysaccharides, nucleic acids, lipids, and proteins that represent up to 98% of the total biomass of the biofilm [66]. The ECM participates during the initial attachment and later provides a controlled extracellular microenvironment. It works as a reservoir of nutrients, debris materials [67], and membrane vesicles [68]; and provides a medium for cell-to-cell communication [69], exchange of genetic information [70], and extracellular enzymatic digestion of nutrients [71]. It also prevents dehydration and inhibits or retards diffusion of antimicrobial factors, which may expose bacteria within the biofilm to sub-inhibitory, or ineffective, concentrations of antimicrobials [72,73]. Biofilm formation can be induced as a protective response to the exposure to sub-inhibitory concentrations of antibiotics. This response has been reported in *P. aeruginosa, Escherichia coli,* and *Staphylococcus epidermidis* among other strains [74,75].

The ECM components of *P. aeruginosa* biofilms have been well studied with alginate, Psl (polysaccharide locus), and Pel (pellicle locus) as three of the principal polysaccharides identified [77]. Alginate is a negatively charged polymer with a role in defining the structure of the biofilm [78]. Alginate was identified in *P. aeruginosa* biofilms isolated from chronic wounds [18]. Mucoid strains have a higher resistance to antimicrobial therapy compared with non-mucoid strains (such as PAO1 and PA14 strains) [79]. Psl is a neutral polysaccharide with a vital role promoting initial bacterial attachment to surfaces and other bacterial cells [80]. Pel is a positively charged polysaccharide that has a close electrostatic interaction with DNA in the ECM network [81]. DNA is another component of the ECM mixture. *P. aeruginosa* produces extracellular DNA (eDNA) composed of both chromosomal DNA and plasmid DNA that can be transferred among the various microorganisms comprising the biofilm [82]. Biofilms could incorporate human DNA from lysate leucocytes into the ECM [72]. 

#### 1.2.2. Phenotypic Tolerance

**Figure 3:**
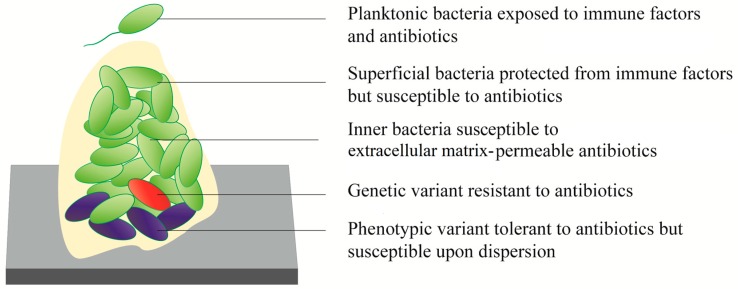
The heterogeneous susceptibility of bacterial biofilms to antibiotics [88].

Biofilms have stratified regions with higher and lower metabolic activity [83]. Approximately 25% of the *P. aeruginosa* biofilm is within an active region due to the proximity to oxygen and nutrient sources [84]. In contrast, bacteria within deeper regions of mature biofilms have minimal metabolic activity [85]. The lower availability of nutrients and a hypoxic environment have been identified as factors that induce bacterial transition into a dormant state [86]. Since most current antibiotics are aimed at disrupting dynamic bacterial processes such as cell division, DNA replication, or protein synthesis, those cells that are not undergoing these processes are not affected by the treatments [87]. These dormant cells are described as *persisters* which represent a phenotypic variant within the population and are another element that contributes to the recurrence observed with biofilm infections [88] (Figure 3). The discovery of these subpopulations in biofilms also helps to explain the low activity observed in antibiotics that can easily penetrate the ECM [89]. It has been demonstrated in vitro that, after a pretreatment with high antibiotic doses that selects for persisters, the surviving cells can recover their original susceptibility to the same antibiotics with the addition of fresh culture media [57]. 

#### 1.2.3. Genetic Resistance

As described previously, *tolerant* bacteria can survive and re-grow after treatment while *resistant* bacteria with active responses can grow in the presence of an antimicrobial [90]. Up-regulation of efflux pumps, reduced affinity of topoisomerases, and deficiencies in outer membrane proteins are some mutational resistance mechanisms identified in *P. aeruginosa* that provide resistance to multiple classes of antibiotics [91]. The close bacteria–bacteria interactions inside biofilms and the exchange of eDNA results in higher mutability compared with planktonically proliferating bacteria [92]. However, these reports of enhanced mutability and resistance contrast with evidence that shows the restored susceptibility of biofilms after dispersion, which does not correlate with the behavior observed in multidrug-resistant strains [93,94,95]. Therefore, the mechanisms of biofilm survival can be generally described by the *tolerance* to antimicrobials followed by the physical protection given by the ECM. Overall, genetic resistance mechanisms seem to have only a minor contribution to the reduced efficacy of antibiotics against *P. aeruginosa* biofilm infections [90]. 

## 2. In Vitro Models to Study Chronic Wound Biofilms

There exists a large variety of systems reported in the literature from static models in microtiter plates to dynamic models of biofilms growing in flow cells. Static systems describe bacterial growth in media that is not diluted during incubation (also called batch mode), which can be stationary or gently shaken as a system to provide a mild shear stress. In dynamic systems, the media is continually refreshed by the flow of sterile media through the culture chamber into a waste container which can be aided by a stirring mechanism. 

### 2.1. Static Models

Static models are closed systems with a finite supply of nutrients. While the nutrients can be replenished at selected time points, there are inherent limits to the possible growth of biofilms over time in these systems. These systems are commonly used in research due to their simplicity and low cost per replicate. Agar and microtiter plates are among the most popular static methods for biofilm study. 

#### 2.1.1. Agar Plates

Agar plates are among the simplest models used in biofilm studies. These models consist of a nutrient-rich gelatinous substrate that provides a finite amount of nutrients to growing bacteria. Agar plates are commonly used in antimicrobial susceptibility testing with the disc- and well-diffusion methods [96], though they have also been used to grow biomass for rheological analysis of biofilms [97,98]. Agar plates are also used in determining the viability, or number of colony forming units, of microorganisms and elucidating variations in colonial morphologies following susceptibility testing [96]. Colony enumeration and morphology analysis are performed following biofilm susceptibility testing in an appropriate model system; however, ECM formation for colonies is minimal and these colonies may not reproduce the ECM structure, which is a hallmark of the biofilm state [99]. 

#### 2.1.2. Colony Biofilms

**Figure 4:**
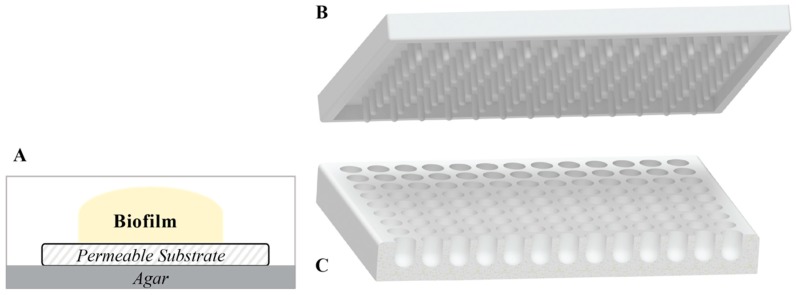
Different systems for static growth of biofilm. (**A**) Colony biofilm model, (**B**) Calgary device lid, and (**C**) round bottom 96-well plate.

These biofilms are developed by the inoculation of sterile membranes filters with a drop of bacterial suspension, while placed on an agar plate [100] (Figure 4A). Heterogenous oxygen and metabolic availability within biofilms of common wound isolates *S. aureus, P. aeruginosa*, and *E. faecalis* were studied using this model [101]. Colony biofilms are grown on a permeable membrane over a semisolid agar surface, which is an advantageous approach, as a semisolid surface is more related to the surface of the infectious site.

#### 2.1.3. Microtiter Plates

Microtiter plates are one of the most popular static model systems for biofilm study as they allow for the screening of a large number of treatments and are useful for the study of the early stages of attachment and biofilm growth. Different sizes of microtiter plates are used to grow biofilms, where the most commonly used are polystyrene 96-well plates. Microtiter plates with round-bottoms are better than traditional flat-bottom plates, as these allow for the homogeneous exposure of the entire biofilm to the growth media and colorimetric reagents [102] (Figure 4C). Tissue-culture treated plates are preferred to prevent biofilm detachment during the rinsing steps. A common problem with this method is the reduced formation of biofilms in the more external wells due to evaporation (i.e., row H and column 1 of a 96-well plate) [103]. Therefore, these wells can be left unused, the plates can be sealed to avoid evaporation-associated problems, or the plates can be maintained in a high-humidity environment. Sealing the plates creates an anaerobic environment, which may be useful for generating oxygen limitations as occurs in deeper layers of clinical biofilms. Oxygen limitation has been identified as a factor that contributes to the enhanced resistance of *P. aeruginosa* biofilm to antimicrobial treatments [84,89,104,105]. An in vitro chronic wound biofilm model was developed by Sun et al. using plates sealed with parafilm [106]. Another critical concern of the microtiter method is the need to prevent excessive disturbance of the biofilm. When using the standard microtiter plate, the biofilm is grown and subsequently, treated within the same plate. Therefore, rinsing steps should be performed carefully so as not to disrupt the biofilm. 

#### 2.1.4. Calgary Biofilm Device

The Calgary device (also known as *MBEC biofilm inoculator*) is a modified microtiter plate that allows for the establishment of equivalent biofilms adhered to removable pegs [107] (Figure 4B). A through-base (equivalent to 12 columns of 8 merged wells) is also available to provide more shear stress during biofilm formation. In this method, the biofilms are established on the pegs, which can be transferred to a new plate with sterile rinsing solution for washing (e.g., PBS) and then into another plate containing the treatment solutions. Transferring the lid instead of rinsing the wells containing biofilms results in less mechanical disruption of the biofilms compared to a traditional microtiter plate system, and therefore allows the growth of older biofilms for up to 12 days [108]. 

### 2.2. Dynamic Models

Dynamic systems may more accurately represent the in vivo biofilm formation in certain disease states, such as in urinary catheters or oral cavity biofilms. Biofilms formed in those conditions are exposed to the shear stresses and nutrient supply of physiological fluids [109]. These models are designed to provide a constant flow of medium over the biofilm-growth surface. The flow can be laminar by dripping the medium on an inclined surface, constant flow using peristaltic pumps, or circular flow obtained by stirring. The flow of the medium, the speed of stirring, and angle of the surface are some of the settings that can be modified in these systems. The effect of flow was studied by Pereira et al., finding that under laminar flow the biofilms had more biomass and were flatter while turbulent flow resulted in less biomass and with more superficial roughness and cell density [110]. These models allow for the development of mature biofilms with large biomasses necessary for accurate rheological and staining analysis.

#### 2.2.1. Flow Cell Systems 

**Figure 5:**
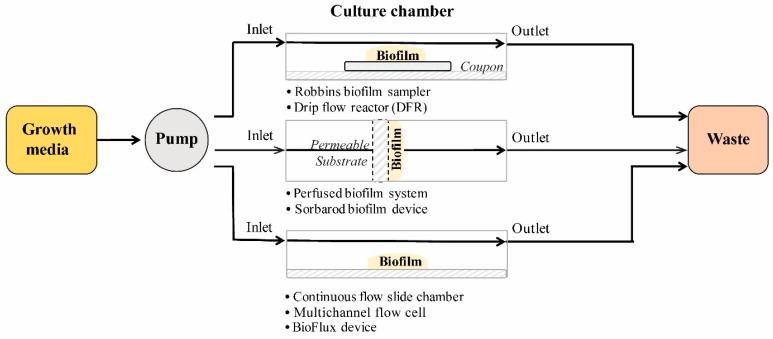
General schematics of the components of flow cell systems for biofilm formation.

In flow cell systems, biofilms grow on tubes, slides, or membranes inside chambers under the flow of medium (Figure 5). The flow is laminar, and the biofilms grow close to the air–liquid interface which allows for a high gas transfer environment [110]. Different surfaces can be used depending on the type of system.

The Robbins biofilm sampler [111] and drip flow reactor (DFR) [112] are flow systems that provide low shear stress and laminar flow in parallel to the biofilm growing surface. In the perfused biofilm fermenter and Sorbarod method, the media diffuses perpendicularly though the biofilm growth surface. In the perfused biofilm fermenter, bacterial aggregation is physically forced by the filtration of a bacterial suspension through a cellulose acetate membrane [113]. In the Sorbarods method, cellulose cylinders (sorbarods) are used as a scaffold for the establishment of biofilm within the cellulose fibers [114,115]. This method provides a substrate for slow growth of biofilms and longer incubation (up to 7 days) due to the larger surface area provided by the scaffold [116]. The continuous flow cells [117] and the BioFlux device are a group of systems designed to allow biofilm formation on glass slides for microscopy. The BioFlux device merges the small scale of a 48-well plate with a microfluidic system to obtain 24 flow chambers compatible with plate readers [118].

#### 2.2.2. Biofilm Reactors

**Figure 6:**
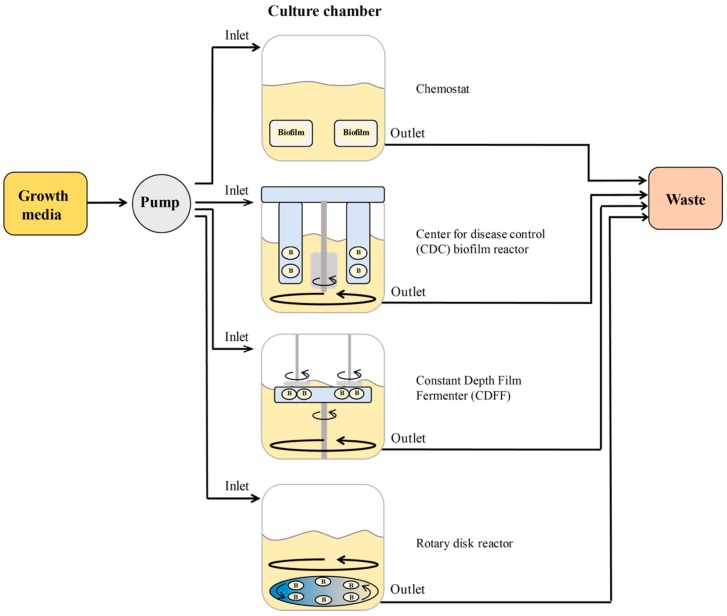
General schematics of the components of biofilm reactors.

A biofilm reactor consists of a vessel containing coupons (surfaces for biofilms formation) submerged in a growth medium. The medium flows from an inlet port to an outlet port. Optionally, circulating flow can be provided by magnetic or mechanical stirring (Figure 6). There is a large variety of coupon materials that can be evaluated in these models. Polycarbonate, stainless steel, hydroxyapatite, titanium, porcelain, and silicone are just a few examples of available coupons for reactors. 

Chemostats were one of the first systems used to study biofilm formation. It comprises a glass vessel with continuous flow from an inlet tubing line that adds media and an outlet line that removes media [119]. The continuous flow of the media provides minimal shear with or without additional stirring elements. The Center for Disease Control biofilm reactor (CBR), the constant depth film fermenter (CDFF), and the rotating disk reactor (RDR) are modified versions of chemostats that include a stirring mechanism and coupon holders. In the CBR, the coupons are fixed with respect to the circulating media, which provides a higher shear compared with the rotating disk reactor [120]. Similarly, biofilms in the CDFF are grown on coupons that are recessed inside sample pans located on a horizontal turntable suspended from the lid of the system. The important aspect of this system is that biofilms can be maintained at a constant depth by the set level of static scraper blades positioned above the coupons [121]. While the CDFF system is frequently used within oral biofilm studies, it has also been used to study biofilms formed from chronic wound isolates [96,122]. The vessel of the RDR contains a rotating disk that holds multiple coupons that rotates aided by a bar creating a low to moderate shear across the surface coupons [123]. 

### 2.3. Advances in In Vitro Biofilm Models

In this section we describe models for infections involving *P. aeruginosa* biofilms, either alone or as multispecies biofilms. Biofilms are grown in systems like those described previously but with the addition of novel elements, such as artificial fluids and mammalian cells, to resemble the physiological conditions of the infection site, which are known to influence the phenotypic expression of *P. aeruginosa* [124]. Table 1 summarizes the models for *P. aeruginosa* biofilm infections reported in the literature. 

#### In Vitro *P. aeruginosa* Biofilm Wound Infection Models

The wound exudate is a rich-fluid used by infectious bacteria as a nutrient source. In vitro models of chronically infected wounds aim to develop biofilms to include relevant wound biofilm microorganisms and to simulate the composition of the wound exudate. Sun et al. studied different growth media and supplements to develop biofilms of the species commonly found in infected wounds [106]. The growth of *P. aeruginosa*, *E. faecalis,* and *S. aureus,* as multispecies biofilm was optimized for the Lubbock chronic wound pathogenic biofilm (LCWPB) system with wound-like media (WLM), made of Bolton broth supplemented with plasma and red blood cells [125]. The group also used this media to study the propagation of anaerobes within multispecies biofilms [126]. Different multispecies-biofilms formed in LCWPB have been used for susceptibility testing [127,128,129,130], to develop in vitro infected agar wound beds [131], and to develop in vivo chronic infection wounds in mice [132]. DeLeon et al. demonstrated the formation of dual *P. aeruginosa* and *S. aureus* biofilms directly suspended in LCWPB [43]. *S. aureus* induces plasma coagulation that serves as a *host-derived* surface for biofilm formation. The dual biofilm growth resulted in higher survival to antibiotics compared to *S. aureus* monoculture. A simpler media consisting of brain heart infusion with plasma was used by Wolcott et al. to grow biofilms of *P. aeruginosa* and *S. aureus* on porcine skin explants [133]. The biofilms reached a maximum resistance after 3 days. *P. aeruginosa* biofilms grown on this explant model were not affected by wiping with moistened gauze; however, the combination of daily cleansing with a surfactant-based wound gel significantly improved biofilm eradication [134]. 

The growth of *P. aeruginosa* within a poloxamer matrix resulted in an enhanced resistance compared with the growth in agar [135]. Biofilms growing within collagen matrices and simulated wound fluid were used to study *P. aeruginosa* and *S. aureus* biofilm formation [136]. The fluid consisted of fetal calf serum, physiological saline solution, and peptone. The distribution of the bacterial aggregates within the collagen matrix correlated with clinical findings where *P. aeruginosa* is found in deeper regions of the wounds while *S. aureus* is located near the surface [18]. The susceptibility of PAO1 biofilms to tobramycin was model-dependent, where the highest reduction in proliferation was observed in LCWPB and the lowest for those in collagen matrices [137]. Bowler and Parsons used gauze as growth supports for *P. aeruginosa* biofilms in their model, which included tryptic soy agar and dried leather to represent the peri-wound skin [138]. The model was developed to evaluate different wound dressings. To evaluate semisolid formulations, such as gels, *P. aeruginosa* biofilms were developed on polyurethane sponges by Martineau and Dosch [139]. 

The colony-DFR biofilm method, combining the cellulose support of the colony biofilm method and a DFR, allowed the development of a multispecies biofilm including the strict anaerobe *Clostridium perfringens* [140]. A cellulose matrix was used by Townsend et al. in their interkingdom model including two bacterial species, *P. aeruginosa* and *S. aureus,* and one fungal species, *Candida albicans* [141]. Triadic biofilms grown in this model had increased biomass and enhanced resistance to antimicrobials compared with those grown in microtiter plates. Crosslinked hyaluronic acid and collagen has also been used as a scaffold to grow *P. aeruginosa* biofilms in an artificial wound model [142].

### 2.4. Summary of In Vitro Model Systems

Multiple systems have been developed to characterize the growth and susceptibility of biofilms to antimicrobials. We described dynamic and static systems where the biofilms are grown under different degrees of shear stress and supply of fresh media. In chronic wounds, the sources of shear stress are represented by the rate of production of wound exudate, which could be considered low. Therefore, among the available systems, those that can be set to minimal flow/stirring may be a better option for in vitro chronic wound biofilm formation.

Microtiter plates are advantageous for in vitro testing compared with biofilm reactors and flow cells, as they allow for high-throughput screening of experimental treatments. An anaerobic environment can be achieved in this system by sealing the plates, which is known to be one characteristic of the biofilm microenvironment, and could better model the response to antibiotics observed in vivo. The limitations of microtiter plates, and other in vitro systems, include the biofilm formation on an abiotic surface, and the need for continuous nutrient supply. However, these models can be upgraded by including other relevant microbial species and simulating fluids, such as wound exudate or simulated wound-like media, as these elements can influence the gene expression of microorganisms within the biofilms and ultimately influence its antimicrobial susceptibility. Three-dimensional (3D) scaffolds for biofilm formation, such as poloxamer and collagen matrices, are especially useful in testing experimental topical treatments for chronic wounds.

## 3. Evaluation of In Vitro Models

Microbial susceptibility to a treatment has traditionally been assessed using planktonic cultures to determine the minimum inhibitory concentration (MIC). As mentioned, this method of assessing susceptibility poorly translates into clinical outcomes. Therefore, a myriad of methods to determine susceptibility of biofilms to treatments have been developed in order to understand the minimum biofilm eliminating concentration (MBEC) [96], also referred to as the minimum bactericidal concentration (MBC) [143]. Various assessments can be used to determine the validity of any treatment for biofilm removal or prevention including colorimetric assays, qualitative microscopic analysis, and mechanical stability tests, among others. The following overview of assays used in the study of in vitro wound biofilm models is arranged according to the general instrumentation and study objective. 

### 3.1. Viability Determination by Colony Counting and Cell Staining

#### 3.1.1. Colony Forming Units

Colony forming units have traditionally been used with planktonic cultures and MIC determination. For biofilms, physical detachment is first used to create a suspension from the biofilm. Direct scrapping of the surface, sonication, and vortexing are used frequently [133]. After scrapping, the surface of the coupon is washed with sterile solution into a beaker to a final known volume before vortex or sonication to homogenize the sample.

Once a homogenous suspension is obtained, it is serially diluted and plated using the spread plate (SP) or the drop plate (DP) methods [144]. In the SP method, a sufficient volume of suspension (about 0.1 to 1 mL) is spread on the surface of an agar plate (one dilution and one replicate per plate). The DP method can be used as an alternative with smaller volumes and the dilutions can be performed in a 96-well plate. The method only uses drops of 10 or 20 µL that are spotted on the agar plate which allows for plating of different dilutions on one plate [145]. The results are commonly reported as the number of colony forming units per area (CFU/cm^2^), per volume (CFU/mL), or reduction with respect untreated biofilms (log reduction) [146,147]. While colony counting remains the standard for determining the viability of microorganisms, it is a labor- and resource-intensive process [148]. A limitation of this method it that it can be difficult to use with multi-species biofilms if selective agar does not exist for a microorganism in the biofilm [149]. Therefore, colorimetric methods, such as the one described below, are more preferable in these conditions.

#### 3.1.2. SYTO 9/PI (Live/Dead BacLight Dye)

SYTO 9 fluorescent dye, which diffuses through cellular membranes and works by binding to nucleic acids in dead and live cells, is suitable for estimating the total number of cells in biofilms [150]. SYTO 9 can be used alone; however, Stiefel et al. found that it stains dead *P. aeruginosa* cells stronger than live cells and may be inadequate for quantifying total cell counts for some strains, especially gram-negative bacteria [151]. An additional disadvantage of SYTO 9 is that it has been shown to photobleach rapidly, especially when used in combination staining with propidium iodide (PI), as discussed below [151]. Alternatives to SYTO 9 such as SYBR green and acridine orange have also been used, but have their own limitations in terms of staining intensity [151].

The Live/Dead BacLight dye, consisting of a combination of SYTO 9 and PI, allows for the visualization and characterization of the biofilm regions affected by antimicrobial agents based on the different membrane permeability of the stains and comes in a convenient ready-to-use kit [152,153]. The components of this technique work by binding to nucleic acids, resulting in the visualization of different colors representing all bacteria (green; SYTO 9) and dead bacteria (red; PI) using confocal laser microscopy. An advantage of using these reagents is the ability to use them with different measurement techniques. These reagents can also be used with spectrophotometers and flow cytometers [154,155]. This assay is typically used with flow cell systems and microtiter plates, but has also been used with CDC biofilm reactor coupons [156]. The addition of PI to SYTO 9 staining has been shown to result in a weaker intensity of *S. aureus* dead cells compared with living cells, but a stronger intensity for *P. aeruginosa* dead cells, demonstrating the displacement of SYTO 9 by PI as intended [151]. SYTO 9/PI stains have been used with *S. aureus* models formed in microtiter plates [150,157,158], collagen [158], and flow cell systems [159]. 

#### 3.1.3. Acridine Orange

Acridine orange (3-N, 3-N, 6-N,6-N-tetramethylacridine-3,6-diamine) is a weak base that intercalates with nucleic acids and can be used to quantify the total number of cells [160]. However, unlike SYTO 9, acridine orange (AO) intensity does not significantly increase when bound to DNA; therefore, unbound acridine orange must be thoroughly rinsed before fluorescence intensity is measured [151]. AO can also be used with PI to determine live and dead cell counts [160]. AO has been used with *S. aureus* and *P. aeruginosa* biofilms grown in microtiter plates [150]. 

### 3.2. Metabolic Activity

#### 3.2.1. Tetrazolium Salts (INT, TTC, CTC, MTT, & XTT)

Tetrazolium salts are commonly used to measure the metabolic activity of bacteria and yeast in biofilms. The electron transport system of metabolically active bacteria causes the reduction of tetrazolium salts forming formazan derivatives that can be measured colorimetrically. TTC (2,3,5-triphenyl-tetrazolium chloride) has been used in agar and media for several decades to selectively isolate *S. mutans* from other Streptococci strains based on the reduction of TTC to red 1,3,5-triphenylformazan (TPF) [161]. Recently, TTC was optimized to determine the metabolic activity of *P. aeruginosa* biofilms in 96-well plate systems [162]. While MTT (3-(4,5-dimethylthiazol-2-yl)-2,5-diphenyltetrazolium bromide) can be used in bacterial studies, this salt is more commonly used in mammalian cell studies because of its reduced solubility compared with XTT (2,3-bis-(2-methoxy-4-nitro-5-sulfophenyl)-2H-tetrazolium-5-carboxanilide inner salt) [163]. MTT is sometimes used to visualize the patterns of metabolic activity within a biofilm after sectioning [164,165]. XTT is more commonly used to study bacterial cells. Most of the XTT-reported methods include a reagent to potentiate the bio-reduction of the tetrazolium salt, such as menadione [162,166] or phenazine methosulphate [167]. Other variables are the incubation time (2–7 h of incubation), and the wavelength of absorbance readings [167]. 

The main advantage of these techniques compared with traditional colony counting is the reduced experimental time. However, there are some limitations including the difficulty in comparing XTT readings across species and in multispecies biofilms [168], these reagents have shown bacterial inhibitory effects and therefore should be used only as end-point assays [169], and because of the mechanism of action, these assays have low sensitivity to detect bacteria in dormant/persistent states [170]. Other forms of tetrazolium salts include 2-(*p*-iodo-phenyl)-3-*p*-(nitrophenyl)-5 phenyltetrazolium chloride (INT) and 5-cyano-2,3-ditolyl tetrazolium chloride (CTC). 96-well plate models have been used with tetrazolium salts to study metabolic activity of *P. aeruginosa* and *S. aureus* biofilms [150,162,171]. TTC has also been used recently with a modified agar system known as the antibiofilm dressing’s activity measurement (A.D.A.M) test of *S. aureus* or *P. aeruginosa* biofilms to assess the anti-biofilm potential of chronic wound dressings [172].

#### 3.2.2. Resazurin (Alamar Blue, PrestoBlue, & CellTiter-Blue)

Resazurin-based assays are dependent on the reduction of resazurin to resorufin for the evaluation of cell growth. The blue non-fluorescent and non-toxic resazurin is reduced to resorufin, a pink and fluorescent dye, by the oxidoreductases within metabolically active cells [173]. Resorufin can also be further reduced to hydroresorufin, an uncolored and non-fluorescent dye. Resazurin-based assays are increasingly being used in biofilm studies as they offer many advantages over traditional tetrazolium-based assays. Resazurin assays are non-toxic to eukaryotic and prokaryotic cells, allowing additional assays to be performed, and consume less time compared with XTT assays, with measurements typically taken after 30–120 min [174,175,176]. 

In addition, there has been shown to be a good correlation between resazurin-based quantification and CFU counts [177]. Initially, lower limits of quantification (>10^6^ CFU/biofilm) needed to adequately detect metabolic activity compared with the background were very high [177]. However, newer techniques involving incubation of resazurin reagent with media and bacteria have decreased the lower limits of quantification to 10^3^ CFU/biofilm [174]. Similarly to XTT, resazurin-based assay protocols may need to be optimized for different cell types and results may be difficult to interpret for multi-species biofilms. Additionally, resazurin-based assays are less resource-intensive than traditional colony counting methods [174]. Resazurin methods have been used with chronic wound-associated microorganisms, *P. aeruginosa*, *S. aureus*, and *Candida albicans*, grown in 96-well microtiter plates [174]. 

#### 3.2.3. Bioluminescence

Adenosine triphosphate (ATP) bioluminescence-based assays can be used to estimate the microbial population of a biofilm, as ATP is the chemical form of energy in all living things [178]. D-luciferin is used in these assays as it undergoes conversion by luciferase to oxyluciferin, a light-generating compound when in the presence of ATP [178]. BacTiter-Glo is a metabolic activity assay kit that is incubated with biofilms for 5 minutes before the luminescence intensity is read. This method is very sensitive at detecting *P. aeruginosa* and *S. aureus* biofilms grown in 96-well plates, with the luminescence signal of the negative control being about 1000 times higher than that of the background [150]. However, the required reagents are expensive compared with other metabolic activity assays. 

Bioluminescence can also be produced with genetically modified strains of bacteria such as PAO1::p16Slux [179] and PAO-1 SEI MCS-5 lite strain [180]. Bioluminescent strains can be used as a metric for viability over time, but are often used simply as an estimate of metabolic activity due to the presence of persister cells that may have reduced signal intensity [180]. Bioluminescent strains of *P. aeruginosa* have been grown in flat-bed perfusion models [181] and microtiter plates [179].

#### 3.2.4. Fluorescein Diacetate (FDA)

Fluorescein diacetate (FDA) assay is based on the capacity of the esterases of live cells to convert non-colored FDA to a yellow fluorescent fluorescein. Peeters et al. found this assay to be more reliable to study the viability of 24 h-old *P. aeruginosa* biofilms, compared with the XTT and the resazurin assays [182]. After the optimization of the assay conditions, a high repeatability and signal among the tested strains was obtained using only 1 h of incubation and 10 mg/mL FDA solution. For this assay, PBS is not recommended due to the high signal obtained in the blank with FDA. Therefore, before the assay, the biofilms need to be rinsed either with MOPS or with BES buffers to remove unadhered cells, but also to remove elements of the growth media that can cleave the FDA reagent and alter the signal [183]. 

### 3.3. Biofilm Biomass and Molecular Stains

#### 3.3.1. Total Biomass: Crystal Violet and Congo Red

Crystal violet (CV) staining is the most common colorimetric method of biofilm quantification. It is used to evaluate the formation of biofilms on a surface as it stains negatively charged molecules and polymers in the extracellular matrix and cells; therefore, it is used for total biomass quantification [125]. Because it stains dead and live cells, it is poorly suited to evaluate the killing of biofilm cells, but can serve to quantify the initial growth of biofilms and prevention capabilities of various treatments [96]. 

The CV solution is added to the wells containing previously-washed biofilms, and after incubation at room temperature the plates are washed and dried [184]. Glacial acetic acid is typically added to dissolve the stained biofilm. This final solution is transferred to a new plate for colorimetric analysis [102,150,185].

Despite its popularity, CV has a number of shortcomings. The primary disadvantage is that numerous washing steps risk the removal of biofilm and overestimation or underestimation of biomass [177]. CV was also shown to be less suitable for quantification of PA biofilms when compared with other species. Peeters et al. found that the signal obtained for two different strains of PA biofilms were considerably lower than the signals for other species such as *Staphylococcus aureus* biofilms [182]. Also, CV was found to be less sensitive for evaluating PA biofilms when compared with tetrazolium salts, and if used, the results should be compared with other assays [162]. In general, CV assays require a relatively large amount of biomass to adequately provide distinction from background staining of a plain microtiter plate [150]. 

Congo red can also be used for quantifying total biomass. The reagent stains the outer membrane of bacteria, as it binds to proteins and polysaccharides [150,186]. Congo red staining has been used with *P. aeruginosa* and *S. aureus* biofilms and mixed chronic wound biofilms. However, due to its high background signal, it is preferable that it be used in biofilm models with abundant biomass, such as those grown in CDFF instead of microtiter plates, to allow for adequate visualization of the biofilm with confocal microscopy [122,150]. 

#### 3.3.2. Cell Components: Safranin and Calcofluor White

Safranin (or safranin red) stain is a non-toxic stain that stains nuclei and mucin. In a study by Ommen et al., safranin staining was reported to result in lower optical densities compared with CV staining, which may indicate that it is not as sensitive for detection of low amounts of biofilm [187]. The authors directly compared safranin staining and CV for *S. aureus*, *S. epidermidis*, and *P. aeruginosa* and found that *S. aureus* formed much less biofilm than the other strains but trends were maintained between CV and safranin stains. Similar results were found by Steifel et al. as well [150]. 

Calcofluor white (CFW) is a non-specific fluorochrome that binds to cellulose and chitin in cell walls and is typically used in imaging fungi, yeasts, and other parasites, but has also been shown to stain β-polysaccharides found in the ECM of bacterial biofilms [188]. CFW is added to biofilms and incubated in the dark at room temperature for a minimum of 15–30 minutes. Then, the biofilms are rinsed and prepared for microscopy. [188]. This stain has been used in a study with chronic wound models initially grown on inserts in a CDFF and frequently used with oral biofilm studies. Biofilm samples were removed from the inserts and fixed to glass slides prior to staining with CFW [122]. CFW can also be used with microtiter plate systems [150]. 

#### 3.3.3. Proteins: SYPRO Ruby and FITC

SYPRO Ruby, a ruthenium complex-based stain, is used for the staining of proteins and has been used with *P. aeruginosa* and *S. aureus* biofilms. An undiluted stain was added to biofilms for 15 minutes before fluorescence intensity was measured using Ex/Em: 460/645 nm [150]. Measurements at Ex/Em: 450/610 nm resulted in insignificant cross-talk with SYTO 9 emission, used for viability determination [188]. The reagent is available in a ready-to-use formulation, which may impede optimization of the stain to novel biofilm models [188]. Additionally, this stain has a high affinity for polystyrene and polypropylene [150], therefore, it is not recommended for biofilms growing on these surfaces, such as microtiter plates. 

Fluorescein isothiocyanate (FITC) staining is similar to SYPRO Ruby staining, as both target proteins in the biofilm. FITC can also target amino-sugars [189]. For staining with FITC, 20 μg/mL of stain was incubated with biofilms for 30 min in the dark, after which the stain was washed twice with 0.9% NaCl solution. Plates were then incubated and vortexed with water to disperse the adhered stain. FITC stains of various concentrations can be made from stocks stored in ethanol, which makes them more amenable than SYPRO Ruby staining in optimizing conditions for specific biofilms [188].

#### 3.3.4. Polysaccharides: Concanavalin A, Periodic Acid–Schiff, and van Gieson

Concanavalin A conjugates bind α-polysaccharides, such as α-D-Mannopyranosyl and α-D-glucopyranosyl residues, found in microbial cell walls [189]. Concanavalin A-Tetramethylrhodamine conjugate (ConA-TMR) has been used with *P. aeruginosa* biofilms [188]. Stiefel et al. tested a ConA-FITC stain with *P. aeruginosa* and *S. aureus* biofilms, staining biofilms for 15 min in the dark [150]. Fluorescently-labeled ConA can be used to bind and visualize alginate in *P. aeruginosa* biofilms [190]. 

Polysaccharides can also be stained using an unspecific marker, Periodic acid–Schiff (PAS) stain. PAS has previously been used with *P. aeruginosa* and *S. aureus* biofilms grown on collagen model systems, though it is more commonly used with fungi [136]. 

Verhoeff-van Gieson stain, like PAS, is a typical histology stain but has been used to stain collagen fibers and bacterial DNA in *P. aeruginosa* and *S. aureus* biofilms grown on collagen model systems [136]. 

#### 3.3.5. Lipids: DiD 

Vybrant DiD cell-labeling solution can be used to stain cell membranes and lipids. In the study of *P. aeruginosa* biofilms, DiD was incubated with the biofilm for 30 min at 37 ˚C before being rinsed and allowing the visualization of accumulated lipids on the PA14 biofilms. Fluorescence intensity was measured with Ex/Em: 644/665 nm [188]. DiD was shown to cross-talk with other fluorophores including ConA-TMR and SYTO 9 [188]. 

### 3.4. Turbidity

Turbidity, or the measurement of optical density, is often used during initializing an in vitro biofilm to select the number of bacteria in the inoculum. This is also the principle behind the McFarland standard, and both use the absorbance of scattered light to measure the concentration of microorganisms in suspension. In general, microbial suspensions are measured at wavelengths of 600–660 nm but may depend on the strain [162,182]. In susceptibility testing, turbidity can also be used to determine the MIC of bacteria in the planktonic phase and in biofilm prevention tests [191]. This measurement has been used to monitor *P. aeruginosa* biofilm dispersion induced by antibiotic treatments [192]. While turbidity can be used to assess growth of biofilms in certain situations, such as with the use of Calgary devices for susceptibility testing of *S. aureus* biofilms treated with magnetic nanoparticles and fields [158], the results should always be compared with additional assays such as viable colony forming units or Live/Dead staining. A number of factors can affect the relationship between absorbance and viable cell counts including cell size, cell shape, interference of pigments in the microorganisms or media, and condition of the instrument [136].

### 3.5. Biofilm Structure 

#### 3.5.1. Fluorescence Microscopy

3D biofilm structure can be visualized using confocal laser scanning microscopy (CLSM) using many of the fluorescent probes discussed in the previous sections. Combinations of fluorescent probes with different Ex/Em filters can be used to visualize different aspects of the biofilm, such as the microorganisms (total/live/dead), polysaccharides, and proteins, among others. In CLSM, multiple 2D focal plane images are taken at different depths and reconstructed to form the 3D structure. This limits out of focus fluorescence signals and allows for resolutions compatible with single cell or biofilm visualization [193]. CLSM can be used to quantitatively assess structural parameters such as biofilm surface area, volume, thickness, and roughness [177].

In addition to the fluorophores already mentioned for use with in vitro assessment of chronic wound associated biofilms, fluorescent in situ hybridization (FISH) probes can be used to study population dynamics of microorganisms in biofilms. The primary difference between the previously mentioned fluorophores and FISH probes is that FISH staining is done with dead, fixed, and permeabilized biofilms [193]. Hybridization probes are single-stranded fragments of DNA or RNA (typically 15–25 nucleotides long) designed to attach to complementary nucleotide sequences associated with various microorganisms within a biofilm [177]. In microbial work, ribosomal RNA is often targeted by the oligonucleotides, as there are a large number of ribosomes in bacterial cells, and this helps achieve a high signal intensity [193]. In order for in situ hybridization of biofilms to occur, cell membranes must be permeabilized using chemical agents, such as detergents. Unbound probes must be washed out before imaging or artifacts are created [193]. 

The main disadvantages of FISH is the necessity of killing microorganisms in the biofilm, specific oligonucleotides to ensure no hybridization of non-target sequences, and detection of metabolically inactive bacteria with few ribosomes [177]. Research into variations in the recognition element has led to new types of FISH, including the popular peptide nucleic acid-based based PNA FISH [136,177]. This method was used in the identification of the multiple biofilm species found in chronic wounds [18].

#### 3.5.2. Raman Microscopy

Raman microscopy (RM) is a non-stain dependent method of assessing the chemical composition and distribution of ECM components and bacteria in biofilms. This is especially valuable for studying components that are not susceptible to known staining techniques. Similar to CLSM, RM can be used with hydrated biofilms, and with the proper equipment can track changes in biofilm chemical composition over time [194]. However, in order to interpret RM spectra, reference spectra of expected components in the biofilm must be taken in order to correctly assign Raman bands to components in the biofilm structure [194]. RM has been used to study changes in polysaccharide, protein, and DNA amounts in the ECM of *P. aeruginosa* planktonic and biofilm cultures [195]. Polysaccharide production in particular was noted to be higher for the biofilm growth mode compared with the planktonic growth mode [195]. Raman has also been used to investigate changes in the chemical structure of *P. aeruginosa* biofilms when treated with different antibiotics. Raman bands associated with DNA and proteins were decreased when compared with the control biofilms [196]. 

#### 3.5.3. Electron Microscopy

Unlike CLSM, traditional scanning electron microscopy is performed on fixed, dehydrated, and coated biofilms [177]. The required dehydration of the sample affects the structure of the biofilm. Therefore, the ECM components are observed as a net lying within the cells, instead of as a hydrogel matrix [197]. Despite this disadvantage, SEM is a powerful technique that allows for a qualitative determination of the cell density in biofilms exposed to different treatments. Environmental SEM (ESEM) is an advantageous microscopy technique for biofilm analysis. It does not require sample dehydration, maintains the biofilm morphology, and therefore requires minimal sample processing [198].

### 3.6. Mechanical Stability

Macro and micro-rheology methods have been used to study the mechanical properties of biofilms [199,200]. Microscopy has been used with biofilms grown in flow cells to determine the effects of different flow rates on *S. aureus* and *P. aeruginosa* biofilms using particle tracking microrheology [201,202]. Atomic force microscopy (AFM) can also be used to measure adhesive and cohesive forces at a sub-nm resolution to better understand the global and local mechanical properties [200]. Single bacteria mechanics and biofilm properties can be assessed using AFM [199]. However, AFM has some drawbacks for biofilm studies, including the effects of cantilever tip shape and chemical properties on measurements and the limitations of isolated bacteria in representing biofilm [199,200]. 

Rheometers have also been used to study the bulk mechanical properties of biofilms. This method typically requires scraping biofilms grown on an agar plate or colony biofilms systems onto the rheometer, or the use of specialized rheometer base systems that allow biofilm growth directly on the rheometer plate [97,203]. Three main bulk rheology tests are commonly performed to assess the mechanical properties of biofilms: creep, stress-relaxation, and dynamic testing [204]. Rheology has been used to study the effects of ECM polymers Pel, Psl, and alginate on *P. aeruginosa* biofilm mechanical toughness [97]. The effect of different phenotypes and biofilm age on biofilm strength have also been assessed using bulk rheology [203].

### 3.7. Zone of Inhibition

Agar plate antimicrobial assays have been used since the initial characterization of penicillin against *S. aureus* [205]. Agar disk-diffusion and agar-well diffusion methods are among the most commonly used [178]. A modified agar-disk diffusion method has been used to determine the zone of inhibition of wound dressings against *S. aureus*, *P. aeruginosa* [155], and *E. coli* [206]. The agar well-diffusion method has been used to study the susceptibility of *S. aureus* and *E. coli* to different concentrations of iron oxide nanoparticles [207]. 

### 3.8. Summary of In Vitro Assays

Traditional methods of measuring susceptibility of microorganisms to chemical treatments were developed for detecting the minimum inhibitory concentration of planktonic cultures. Colony forming unit viability assays remain as the standard for determining microbial susceptibility despite the time consuming and resource-intensive nature of the method. The development of new chemical and mechanical assays and the understanding that biofilms are the predominant growth form of bacteria has led to the development and popularity of new assays for determining biofilm susceptibility (Table 2). Crystal violet is one of the most common assays used to detect biofilm formation and changes in biomass due to treatments. While cautions need to be taken to not disrupt the biofilm when preparing crystal violet assays, the method is simple, inexpensive, and widely accepted for biofilm studies. SYTO 9/PI (Live/Dead) staining is also commonly used to assess the viability of microorganisms in biofilms. While the reagents for this assay are typically more expensive than the simple materials needed for colony counting, the Live/Dead assay allows for quicker quantification with fluorescence spectroscopy. The Live/Dead stain can also be used with confocal laser scanning microscopy and other dyes to visualize the 3D structure of the biofilm. Metabolic activity assays, especially XTT and PrestoBlue, are quick spectroscopic methods that are less expensive than the Live/Dead stain and measure the metabolic activity of microorganisms. Luminescence is another metabolic activity indicator that can be used to measure time-kill curves. Newer methods for determining the effect of treatments on biofilms have focused more on changes to the ECM matrix. Raman microscopy and confocal laser scanning microscopy can be used to map the hydrated biofilm structure, while rheometry can be used to measure changes in biofilm mechanical stability. Many assays have been used with in vitro chronic wound models to better understand different aspects of chronic wound biofilms and the effects of chemical treatments. Here, we have provided a brief overview of many of these methods with a focus on viability, metabolic activity, biomass, structural, and mechanical assays in order to help in the selection of relevant assays for future studies. 

## 4. Conclusions

The field of biofilm research has increased tremendously as new evidence has confirmed the role of biofilms in different disease states including some that previously had not been considered as infectious diseases. Despite the advances in in vitro modeling of biofilm infections, there is still a lack of transferability of promising findings to applications at the clinical level. It is important to continue developing advanced in vitro models and assays to better understand the mechanisms of *P. aeruginosa* and *S. aureus* infections, and to develop novel treatments and more affordable models. We hope that our review can provide the concepts to move forward from the culture of mono-species biofilms on abiotic surfaces towards more complex and relevant models for chronic wounds.

## Figures and Tables

**Table 1:** Advances in models to study *Pseudomonas aeruginosa* biofilms in vitro.

Model	Strains	Support	Surface	Media	Incubation	Application	References
Chronic Wound Infection Models							
Germ carrier model	PA ATCC 27317	Petri dish	Polyurethane sponge	TSB	4, 7, and 24 h	Biofilm susceptibility in in vitro to semisolid formulations	Martineau, 2006 [139]
Poloxamer biofilm	PA NCIMB 8626	Petri dish	Poloxamer hydrogel	MHB	24 h	Biofilm formation and susceptibility testing of different commercial silver-containing dressings.	Percival, 2007 [135]
Lubbock chronic wound pathogenic biofilm (LCWPB)	PAO1, *E. faecalis,* and *S. aureus*	Microtiter plate	Polystyrene 96-well plate	WLM media: 45% Bolton broth, 50% bovine plasma, and 5% freeze-thaw laked horse red blood cells	Anaerobic for 24 h	Definition of media composition for multispecies biofilm formation and susceptibility testing	Sun, 2008 [106]
Porcine explant wound biofilm model	PAO1 and *S. aureus*	TSA plate	Partial thickness wound beds in fresh porcine skin explants	TSB	Up to 5 days	Biofilm susceptibility testing	Wolcott, 2010 [133]
Collagen wound biofilm	PAO1 and *S. aureus*	Culture slides	Collagen matrices	50% fetal calf serum and 50% physiological NaCl in 0.1% Peptone	48 h	Biofilm formation within collagen matrices and susceptibility testing	Werthen, 2010 [136]
Polymicrobial colony-DFR wound biofilm	PA, MRSA, and *C. perfringens* wound isolates	DFR	Polycarbonate membrane	100% BHI with 5% adult bovine serum	3 days	Biofilm formation and susceptibility testing of dressings	Woods, 2012 [140]
Cellulose interkingdom biofilm wound model	PA14, *S. aureus,* and *C. albicans* SC5314	N/D	Cellulose matrices on top of hydrogel	PBS	24 h	Biofilm formation within cellulose matrices and susceptibility testing	Townsend, 2016 [141]
Artificial wound model	PAO1 and clinical isolates	Microtiter plate	Hyaluronic acid and collagen scaffold	Bolton broth with 50% bovine plasma and 5% freeze-thaw laked horse blood	16 h	Biofilm formation and susceptibility testing of antimicrobial peptides	Grassi, 2019 [142]

N/D: Not described; LCWPB: Lubbock chronic wound pathogenic biofilm model; PA: *Pseudomonas aeruginosa*; TSB: tryptic soy broth; MHB: Mueller–Hinton broth, WLM: wound-like media, TSA: tryptic soy agar, BHI: brain heart infusion media; DFR: drip flow reactor; MRSA: methicillin-resistant *Staphylococcus aureus*; PBS: phosphate saline buffer.

**Table 2:** Methods for Biofilm Analysis used with *P. aeruginosa* and *S. aureus* biofilms in vitro.

Category of Evaluation	Principle/Target	Method Overview	Detection Method	Example Detection Settings	Example Model Systems	References
**Viability**						
Colony counting	Viable cells are able to form colonies when plated on appropriate agar substrates	Dispersions of cells are spread or drop-plated. Colonies formed counted after appropriate growth period	Visual		Agar plate	[136,180,208]
SYTO 9	All Cells	Nucleic acids stained and visualized (M) or dispersed by vortexing in appropriate media (S)	FS, FM	Ex: 485 nm Em: 528 nm	Microtiter plate	[150]
SYTO 9/PI	All cells (SYTO9) Dead/membrane permeable cells (PI)	Nucleic acids stained and visualized (M) or dispersed by vortexing in appropriate media (S)	FS, FM	Ex: 485 nm Em: 528 or 645 nm	Microtiter plate, flow cell, collagen model	[150,157,158,159]
Acridine orange	All cells (nucleic acids)	Nucleic acids stained and dispersed by vortexing in appropriate media	FS	Ex: 485 nm Em: 528 nm	Microtiter plate	[150]
Ethidium bromide	DNA	DNA stained and visualized. Appears orange when excited	FS, FM	Ex: 210 or 285 nm Em: 605 nm	Constant depth film fermenter (CDFF) and glass microscopy slide	[122]
Ziehl carbol fuchsin	Bacterial cells	Stains bacterial cells red/purple	LM		CDFF and glass microscopy slide	[122]
DAPI	DNA	DNA stained and visualized.	FM, LM	Ex: 350 nm Em: 470 nm	Microtiter plate and glass slide	[136]
**Metabolic Activity**						
Tetrazolium Salts (INT, TTC, CTC, XTT, and MTT)	Reduction of Tetrazolium to formazan	Dissolved dye from stained biofilms recovered and quantified	AS	INT: 470 nm TTC: 405, 450, 490, 540 nm XTT: 450-492 nm (486nm)	Microtiter plate, modified agar plate	[150,161,162,172]
Resazurin (Alamar Blue, PrestoBlue, CellTiter-Blue)	Reduction of Resazurin to resorufin	Reagent incubated with media and biofilms	FS, AS	Ex: 560 nm Em: 590 nm Abs: 570 and 600 nm	Microtiter plate	[173,174,208]
Bioluminescent Assay (BacTiter)	Catalysis of ATP and luciferin by luciferase	D-luciferin is used in these assays as it undergoes conversion by luciferase to oxyluciferin a light generating compound when in the presence of ATP	L*S		Microtiter plate	[150,179,180,181,209]
Fluorescein diacetate (FDA)	Cleavage of acetate by intracellular esterases	FDA converted to yellow fluorescent fluorescein	FS	Ex: 494 nm Em: 518 nm	Microtiter plate	[182]
**Biomass**						
Crystal violet	Stains negatively-charged molecules and polymers. All biomass (live, dead, and matrix)	Stained biofilms dissolved in appropriate solvent	AS	550 - 600 nm	Microtiter plate	[102,185]
Congo red	Polysaccharides and cell membranes	Stained biofilms dissolved in appropriate solvent	AS	500nm	Microtiter plate CDFF and glass microscopy slide	[122,150]
Safranin	Nuclei and mucin red	Stained biofilms dissolved in appropriate solvent	AS	~535 nm	Microtiter plate	[150,187]
Calcofluor white	beta-polysaccharides in matrix	Biofilms stained and visualized (M) or dispersed by vortexing in appropriate media (S)	FS, AS	Ex: 360, 365, or 400 nm Em: 460, 435, 410 nm	Microtiter plate CDFF and glass microscopy slide	[122,150,188,189]
SYPRO Ruby	proteins	Biofilms stained and dispersed by vortexing in appropriate media	FS	Ex: 450 or 460 nm Em: 610 or 645 nm	Microtiter plate	[150,188]
FITC	proteins and amino-sugars	Biofilms stained and visualized (M) or dispersed by vortexing in appropriate media (S)	FS, FM	Ex: ~488 nm Em: 500-550 nm	Microtiter plate	[150,188,189]
Concanavalin A (Con A) conjugates	alpha-Mannopyranosyl and alpha-glucopyranosyl sugars	Biofilms stained and visualized (M) or dispersed by vortexing in appropriate media (S)	FS, FM	Ex: 543 nm Em: 550-600 nm		[189]
FITC-Con A	polysaccharides	Biofilms stained and visualized (M) or dispersed by vortexing in appropriate media (S)	FS, FM	Ex: 485 nm Em: 528 nm	Microtiter plate	[150]
Con A - Tetramethylrhodamine	Alpha polysaccharides	Biofilms stained and visualized (M) or dispersed by vortexing in appropriate media (S)	FS, FM	Ex: 555 nm Em: 580 nm	Biofilm reactor	[188]
Periodic acid-Schiff (PAS)	Stains polysaccharides		LM		Microtiter plate and glass slide	[136]
van Gieson	Stains collagen Fibers and Bacterial DNA		LM		Microtiter plate and glass slide	[136]
Vybrant DiD	Lipids and Membranes	Biofilms stained and visualized (M) or dispersed by vortexing in appropriate media (S)	FM	Ex: 644 nm Em: 665 nm	Biofilm reactor	[188]
**Turbidity**						
Turbidity threshold method	Quantification of dispersed cells	Measure absorbance of bacterial suspension and bacteria-free media and compare to a known dilution series	AS	600 nm	Microtiter plate	[150,209]
MacFarland standards	Quantification of dispersed cells	Measure absorbance of bacterial suspension and McFarland Standards (mixtures of H_2_SO_4_+BaCl_2_ or latex particles)	AS	625 nm	Microtiter plate	
**Structure**						
Scanning electron microscopy (SEM)	Visualization of morphology and distribution of microorganisms and extracellular matrix (ECM)	Biofilms typically fixed and negatively stained (SEM)	SEM/Cryo-SEM/ESEM	Varies by instrument	Flat-bed perfusion system, collagen model	[158,181,209,210]
Confocal scanning laser microscopy (CLSM)	Isolation of 3D microbial community	Use applicable stains and dyes listed above to visualize various aspects of the biofilm	FM	Varies by stain/dye	Glass microscopy slide, flow cell	[209,211]
Fluorescent in-situ hybridization (FISH/PNA-FISH)	Visualize patterns of microbial colonization	Fluorescently labeled oligonucleotide probes hybridize to ribosomal RNA in cells that have been fixed and permeabilized	FM	Varies by stain/dye	Glass microscopy slide	[136,177,209]
Raman microscopy	Mapping of microorganisms and ECM Raman spectra		RM	Varies by instrument/target	Raman-neutral slide	[209]
**Mechanics**						
Atomic force microscopy (AFM)	Mapping of local and global adhesive and cohesive forces	Measure force-displacement curves		Varies by instrument/target		[97,212]
Micro-rheology		Measure behavior of isolated bacteria under different physical conditions	FM, LM	Varies by stain/dye	Flow cell	[202]
Bulk rheology	Biofilms have viscoelastic properties	Measure viscoelastic properties of ECM matrix with microorganisms	Rheometer	Varies by instrument	Agar plate, colony system	[97]
**Other**						
Agar disk/well-diffusion	Zone-of-inhibition of therapies measured	Agar plates inoculated with bacteria are exposed to a therapy within a defined area. Following growth period, area of new growth measured	Visual		Agar plate	[178]

Analysis: PI: propidium iodide; DAPI: 4′,6-diamidino-2-phenylindole; INT: 2-(p-iodo-phenyl)-3-p-(nitrophenyl)-5 phenyltetrazolium chloride; TTC: 2,3,5-triphenyl-tetrazolium chloride; CTC: 5-cyano-2,3-ditolyl tetrazolium chloride; XTT: 2,3-bis-(2-methoxy-4-nitro-5-sulfophenyl)-2H-tetrazolium-5-carboxanilide; MTT: 3-(4,5-dimethylthiazol-2-yl)-2,5-diphenyltetrazolium bromide; FITC: fluorescein isothiocyanate; PNA: peptide nucleic acid. Detection Methods: M: Microscopy; S: Spectroscopy; F: Fluorescence; A: Absorbance, L: Light; L*: Luminescence; R: Raman; Ex: Excitation wavelength; Em: Emission wavelength.
